# Computer vision syndrome, musculoskeletal, and stress-related problems among visual display terminal users in Nepal

**DOI:** 10.1371/journal.pone.0268356

**Published:** 2022-07-19

**Authors:** Amar Das, Sangam Shah, Tara Ballav Adhikari, Basanta Sharma Paudel, Sanjit Kumar Sah, Rakesh Kumar Das, Chiranjiwi Prasad Shah, Pragati Gautam Adhikari

**Affiliations:** 1 Maharajgunj Medical Campus, Institute of Medicine, Tribhuvan University, Maharajgunj, Nepal; 2 Department of Public Health, Section for Global Health, Aarhus University, Aarhus, Denmark; 3 Tribhuvan University Teaching Hospital, Maharajgunj, Nepal; 4 Department of Ophthalmology, Institute of Medicine, Tribhuvan University, Maharajgunj, Nepal; Hanyang University, REPUBLIC OF KOREA

## Abstract

**Background:**

The use of computers and other Visual Display Terminal (VDT) screens is increasing in Nepal. However, there is a paucity of evidence on the prevalence of Computer Vision Syndrome (CVS) and other occupational health concerns among employees working in front of VDT screens in the Nepalese population.

**Objectives:**

This study aims to estimate the prevalence of CVS, musculoskeletal and work-related stress among VDT screen users in the office, as well as their understanding and usage of preventive measures.

**Methods:**

The study was a cross-sectional descriptive study among 319 VDT users in office settings in Kathmandu Metropolitan City, Nepal, using a semi-structured self-administered questionnaire. Multivariate logistic regression analysis was conducted to identify the associated factors at 95% CI. P-value <0.05 was considered as statistically significant.

**Results:**

The prevalence of CVS was 89.4%. More than eight out of ten study participants reported at least one visual and musculoskeletal symptom. Work-related stress, which was moderate-difficult to handle, was present in 36.7% of the study population. The mean±SD computer usage per day was 7.9±1.9 hours. Tired eye (63.3%), feeling of dry eye (57.8%), headache (56.9%) were the common visual symptoms of CVS reported. Total computer use/day > = 8 hours OR 2.6, improper viewing distance OR 3.2, Not using an anti-glare screen OR 2.6, not using eye-drops, and not wearing protective goggles OR 3.1 were significantly associated with the presence of CVS. There was no statistically significant association between visual symptoms of CVS, musculoskeletal symptoms, and stress with gender.

**Conclusion:**

CVS was substantially related to not employing preventive measures, working longer hours, and having an incorrect viewing distance. With more hours per day spent in front of a VDT screen, work-related stress and musculoskeletal complaints were also found to be important correlates. Similarly, work-related stress was found more among those who had less than five years of job.

## Introduction

Computers have become the most ubiquitous and indispensable office tool. Its need for eyesight is high, in contrast to other office equipment (telephone, written or printed page). Computer users may get computer vision syndrome (CVS) as a result of this [1, 2]. CVS affects computer users from all walks of life, making it an important public health issue and a potential occupational epidemic of the twenty-first century [3, 4]. CVS is defined as “a complex eye discomfort and vision problems such as eye strain (fatigue), blurred vision, excessive tearing, double vision, headache, light sensitivity, dry eye, and irritated eye experienced while using computers for longer periods of time [5, 6],” according to the American Optometric Association. The symptoms include eye redness, eye strain, burning sensation, blurred vision, gritty sensation, headache, and neck pain.

Users’ health effects can be measured in terms of stress, postures, health performance, and productivity [7], as well as aesthetic comfort [8]. This leads to decreased productivity, higher error rates, worse job satisfaction, and a lower quality of life [9, 10]. According to a study, productivity can be reduced by as much as 40% [11]. Every year, a million new cases of CVS are diagnosed around the world. According to the Seattle Times Company, up to 70 million workers globally are in danger of developing computer vision sickness, and this figure is expected to rise [12]. According to the findings, 70% to 90% of persons who use computers frequently, whether for work or leisure, show one or more symptoms of computer vision syndrome [12].

Currently, there is a growth in digital work in all sectors in Nepal, and computers are offered to all professional staff without regard for the health consequences of using them. All of those employees working on the computer are at a higher risk of developing eye issues than other employees who are not working. Stress and musculoskeletal disorders are prevalent among visual display terminal users. In two studies, working and studying for most of the day in front of a computer while sitting in a chair has resulted in a slew of other health issues, including stress (35% & 44%) and musculoskeletal difficulties (77.5% & 63%) [13, 14].

As a result, the purpose of this research is to describe the prevalence of CVS and its associated personal characteristics among Nepalese IT professionals. It closes a study gap by taking into account all personal aspects and conducting measurements. This research will also look into the prevalence of other workplace issues such as musculoskeletal ailments and work-related stress. The findings of this study can help ophthalmic experts, health care providers, and other training institutions plan, design, and revise training curricula to improve employees’ knowledge and awareness of CVS.

## Methodology

### Ethical approval

Ethical approval was obtained from the research ethics committee of the Institutional Review Committee (IRC) of the Institute of Medicine (IOM). Official letters of cooperation from IRC were written to respective study districts and companies. Written informed consent was obtained from all study subjects to allow the use of anonymous personal and clinical data in research. Confidentiality of the information was maintained thoroughly by de-identification.

### Study design

An institution-based cross-sectional study was employed to assess the prevalence and factors of CVS, stress, and musculoskeletal among IT workers in Kathmandu valley, Nepal.

### Study setting

This study was conducted in the two IT software companies, two offices, and one bank situated in Kathmandu valley, Nepal. The study was conducted from March 2021 to May 2021.

### Study participants

The study population was all employees of IT software companies, offices, and bank of Kathmandu valley in all (three districts) who are computer. All the employees who are working in the above-mentioned sites of Kathmandu valley were taken as the study population.

#### Inclusion criteria

The inclusion criteria included all workers who used computers to complete their tasks for at least 4 hours per day during the working days and had worked using the computer for at least 12 months.

#### Exclusion criteria

The exclusion criteria included workers who worked less than 4 hours in front of a computer per day and less than 12 months.

### Sampling

The researcher intends to perform the study among employees of enterprises and banks, taking into account their age, amount of computer exposure, number of employees and customers, and resource and time constraints. The companies were chosen at random using simple random sampling (lottery method), considering the sample’s representativeness and logistical issues. Using proportionate stratified random selection, 340 office workers from 5 offices were randomly picked without replacement from a full list of respondents acquired from each company’s human resource department. Each firm was treated as strata in this sampling technique, and the number of samples was proportional to the population for each stratum. Then, using Microsoft Excel and random numbers, samples were taken from each office using a simple random sampling without replacement approach. 21 of the participants were excluded as they either did not fill the questionnaire or the questionnaire they filled was incomplete.

### Study tools

#### Pretested structured questionnaire

A set of experts validated self-administered questions were used to collect socio-demographic data, symptoms of CVS, stress, and musculoskeletal disorders. (S1 File) Questions on symptoms of CVS were adapted from a previous study done by Shrestha et al. [15]. Nordic questionnaire [16] was used for musculoskeletal disorder symptoms that has specificity of 88% and sensitivity of 92% while work related stress questionnaire [17] was used for assessing stress. The presence of blurred vision, eye strain, and eye fatigue, redness of eyes, watery eyes, eye dryness, double vision, eye irritation, burning sensation, and headache were assessed as symptoms of CVS. The presence of at least one symptom, either intermittently or continuously, during the previous 12 months in one or two eyes was considered as presence of CVS [18]. The questionnaire was prepared first in English and translated to Nepali (the national language of the study area) to make the data collection process simple. To check its consistency in the meaning of words and concepts, the questionnaire was translated back to the English by language experts after the data collection. The tools adapted in this study have been previously used in a similar setting and similar study population in Nepal. For instance, the symptoms of CVS tools were used by Shrestha et al. [15]. Similarly, Nordic questionnaire for musculoskeletal disorder were used among canteen staff and agricultural farmers by Shakya et al, and Mahto et al respectively [19, 20].

### Study variables

Visual, musculoskeletal symptoms and work-related stress were only taken into account if they were present for at least seven days per year.

#### CVS symptoms

Those who had CVS were also divided into two groups. Participants with seven out of nine symptoms or a grade of four or five for more than two symptoms were classified as having "severe CVS," while the others were classified as having "mild-moderate CVS."

#### Musculoskeletal disorder

A standardized Nordic questionnaire was used for complaints of musculoskeletal disorder.

#### Work-related stress questionnaire

The study population was divided into three groups based on the results of a work-related stress questionnaire: i) those who could handle work-related stress easily, ii) those who could handle it reasonably well, and iii) those that needed some kind of help to relieve the strain.

#### Statistical analysis

Data was gathered using a printed questionnaire as well as an internet tool called Google Forms. MS-EXCEL 2010 was used to enter the data, while SPSS version 26 was used for statistical analysis. Tables, charts, figures, and statistical tools were used to present the study’s findings. The chi-square test was used to examine the relationship between categorical factors and dependent variables (CVS, musculoskeletal complaints, and work-related stress). Gender, number of hours per day spent in front of a computer, and number of years in present job was among the categorical variables. The chi-square test was also used to evaluate the relationship between variables such as the usage of various preventive measures, the distance between the screen and the computer, and the dependent variable (CVS). For all of the analyses, a P-value of 0.05 at a 5% level of significance was considered statistically significant. In addition, odds ratios and 95% confidence intervals were determined for categorical variables that had a significant relationship with the presence of CVS.

## Results

### Characteristics of study participants

A total of 319 office workers that participated in the study and provided complete information. The study group’s average age was 33.4±9.4 years. More than six out of ten participants were between the age group of 20 to 35 years, with 71.6% of the participants being male. The average time spent on the computer was 7.9±1.9 hours, with 68.8% using it for 7–9 hours. The average length of job was 7.1±7.2 years, with 57.8% of them working for more than five years. Right-handed employees made up 92% of the participants in our study. Table 1 shows the characteristics of the study participants.

**Table 1:** Characteristics of the study participants.

Variables	Total n (%)	Male n (%)	Female n (%)
**Age**			
20–30	140(44.1%)	73(32.1%)	67(73.6%)
31–40	123(38.5)	105(46.1%)	18(19.8%)
41–50	39(11.9%)	33(14.5%)	6(6.6%)
>50	17(5.5%)	17(7.3%)	-
**Number of years worked**			
0–5	184(57.8%)	102(44.7%)	82(90.1%)
6–10	38(11.9%)	38(16.6%)	-
11–15	44(13.8%)	41(17.9%)	3(3.3%)
16–20	32(10.1%)	32(14.2%)	-
21–25	21(6.4%)	15(6.6%)	6(6.6%)
**Number of hours/days**			
<7 hours	59(18.3%)	44 (19.3%)	15(16.5%)
7–9 hours	219(68.8%)	158 (69.3%)	61(67.0%)
>9 hours	41(12.8%)	26 (11.4%)	15(16.5%)
Total	319 (100%)	228 (71.5%)	91 (28.5%)
Right and left handed worker			
Right handed	293 (92%)	284 (97%)	9 (3%)
Left handed	26 (8%)	16 (61.6%)	10 (38.4%)

### Prevalence of computer vision syndrome

Table 2 displays the prevalence of each symptom. At least one symptom of CVS was reported by 84.4% of participants. There was no discernible link between gender and the incidence of CVS. The most common symptoms mentioned were tired eyes (63.3%), dry eyes (57.8%), headaches (56.9%), and pain behind the eyes (45.9%). Males had significantly greater rates of headache, dry eyes, blurred vision, watery eyes, burning sensations, and redness of the eyes (p-value <0.05), but no other visual symptoms were associated with gender. Those with mild-moderate CVS symptoms accounted for 58.7% of those with CVS, whereas the remaining 41.3% had severe CVS symptoms.

**Table 2:** Visual symptoms in study participants.

Symptom	Total	Male**	Female**	p-value
Tired eye	202 (63.3%)	62.7%	64.8%	P = 0.723 (NS)
Dry eyes	184(57.8%)	61.4%	48.4%	P = 0.033*
Headache	181(56.9%)	53.1%	65.9%	P = 0.036*
Pain behind eyes	146(45.9%)	43.9%	50.5%	P = 0.279 (NS)
Itchy eyes	140(44.0%)	40.8%	51.6%	P = 0.078 (NS)
Blurred vision	129(40.4%)	44.3%	30.8%	P = 0.026*
Watery eyes	129(40.4%)	49.1%	18.7%	P = 0.00001*
Burning eye	117(36.7%)	40.8%	26.4%	p<0.016*
Eye redness	114(35.8%)	45.6%	11.0%	P = 0.00001*

*chi square test

** % denotes number of (male/female) having the symptom/ (male/female)

NS- not significant

### Use of preventive measures

Around 51.3% of the population did not take any preventative steps. Ultraviolet protective goggles were used by 30.3% of persons, anti-glare screens were used by 15.3%, and artificial tear drops were used by 26.6%. 62.4% of people maintained a 40-70cm viewing distance. Table 3 shows the study population’s adoption of preventive measures and the distance maintained between the VDT screen and their eyes.

**Table 3:** Use of preventive measures in study participants.

SN	Preventive measures	Number (%age)
1	Use of Eye drops	85(26.6%)
2	Use of anti-glare screen	49(15.4%)
3	Use of protective goggles	97(30.3%)
4	Distance between eye and screen:- <40 cm	79(24.8%)
	40–70 cm	199(62.4%)
	>70 cm	41(12.8%)

### Factors associated with CVS

The results of the regression analysis between CVS and other study variables are shown in Table 4. Total computer use/day > = 8 hours [OR **=** 2.51; CI (1.29, 4.89) p = 0.007], improper viewing distance [OR = 2.61; CI (1.17, 5.82) p = 0.019], not wearing protective goggles [OR = 2.38; CI (1.17, 5.82) p = 0.017] not using eye-drops [OR 2.28; CI (1.17, 4.43) p = 0.015] and not wearing protective goggles [OR 2.38; CI (1.17, 5.82) p = 0.017] were found to have a significant association with the presence of CVS (p<0.05) while use of antiglare screen had no significant association with the prevalence of CVS.

**Table 4:** Association between CVS and study variables.

Variables	Categories	Bivariate analysis			Multinomial-Logistic-regression		
		CVS-yes Total (%)	CVS-no Total (%)	p-Value	Odds Ratio	95% CI	p-Value
Total computer use/day	> = 8 hours	170 (89.4%)	20 (10.6%)	<0.05^*^	2.51	(1.29, 4.89)	0.007
	<8 hours	99 (76.7%)	30 (23.3%)			-	
Distance between screen and eye	Proper(40-70cm)	158 (79.4%)	41 (20.6%)	<0.05^*^	2.61	(1.17, 5.82)	- 0.019
	Improper	111 (92.5%)	9 (7.5%)				
Anti-glare screen	Yes	35 (71.4%)	14 (28.6%)	<0.05^*^	-	-	-
	No	234 (86.7%)	36 (13.3%)		-	-	-
Wearing protective goggles	Yes	71 (72.9%)	26 (27.1%)	<0.05^*^	-	-	-
	No	198 (89.2%)	24 (10.8%)		2.38	(1.17, 5.82)	0.017
Use of eye drop	Yes	61 (71.8%)	24 (28.2%)	<0.05^*^	- 2.28	- (1.17, 4.43)	- 0.015
	No	208 (88.9%)	26 (11.1%)				

*chi square test

### Prevalence of musculoskeletal symptoms

In 80.7% of the research participants, at least one of the musculoskeletal complaints was present. The most common symptoms mentioned were pain in the lower back (61.5%), neck (45.2%), and shoulder (39.4%). 76.1% of patients had difficulty with their low back, shoulder, or neck. Table 5 shows the prevalence of visual symptoms, musculoskeletal complaints, and work-related stress by gender, the number of years worked in present employment, and hours spent in front of the VDT screen.

**Table 5:** Prevalence of CVS symptoms, musculoskeletal symptoms and work-related stress.

	Visual symptoms (CVS)	Musculoskeletal symptoms	Work related stress
	N (%)	N (%)	N (%)
Gender*			
Male	196(72.8%)	178(69.3%)	151(72.7%)
Female	73(27.2%)	79(30.7%)	57(27.3%)
No. of years working^#^			
0–5	152(56.5%)	143(55.7%)	125(59.9%)
6–10	32(11.9%)	35(13.6%)	21(10.1%)
11–15	41(15.3%)	41(15.9%)	36(17.6%)
16–20	26(9.8%)	23(9.1%)	10(4.7%)
21–25	18(6.5%)	14(5.7%)	16(7.9%)
Hours/Day in front of VDTs^$^			
<7 hours	50(18.5%)	38(14.8%)	47(22.8%)
7–9 hours	181(67.4%)	178(69.3%)	130(62.5%)
>9 hours	38(14.1%)	40(15.9%)	31(14.7%)
**Total**	**84.4%**	**80.7%**	**36.7%**

* p-value>0.05 for gender and different symptoms (Not significant)

^#^p-value<0.05 for number of years working in current job and musculoskeletal problem, work related stress

^$^p-value<0.05 for Hours/Day in front of VDTs and musculoskeletal problem, work related stress

**Fig 1:**
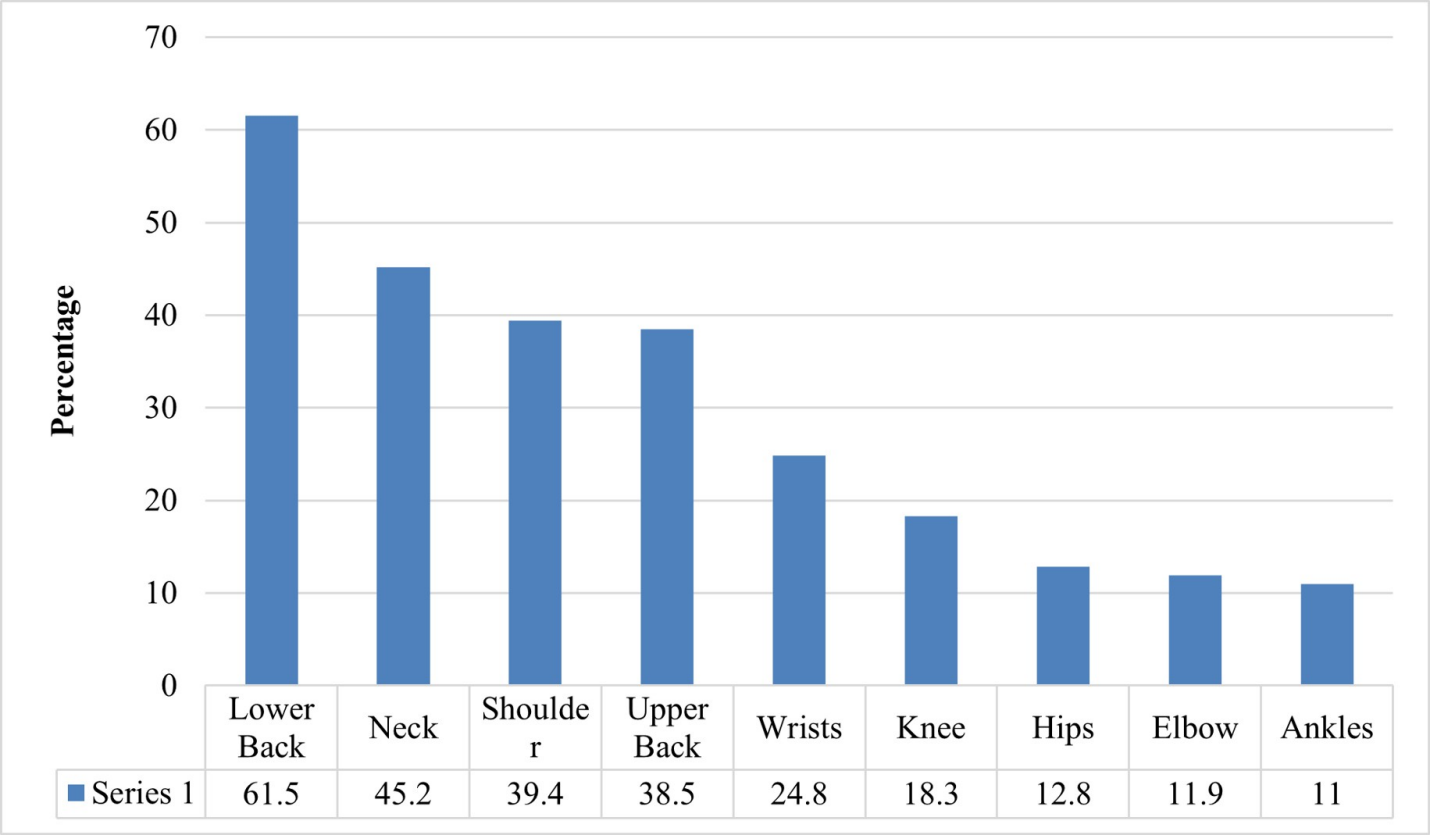
Site of musculoskeletal discomfort in study participants.

**Fig 2:**
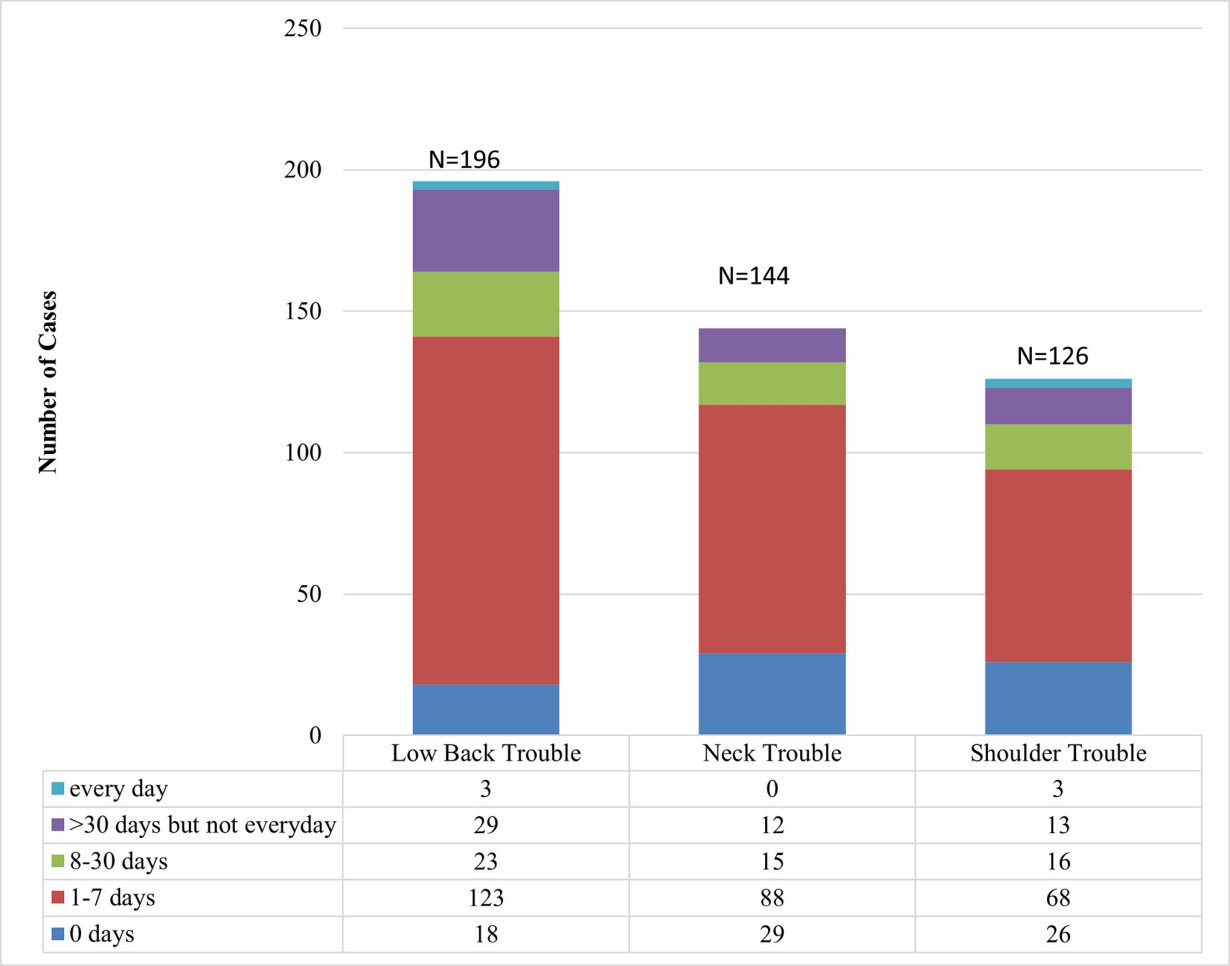
Number of days’ study participants experiencing musculoskeletal problem.

**Fig 3:**
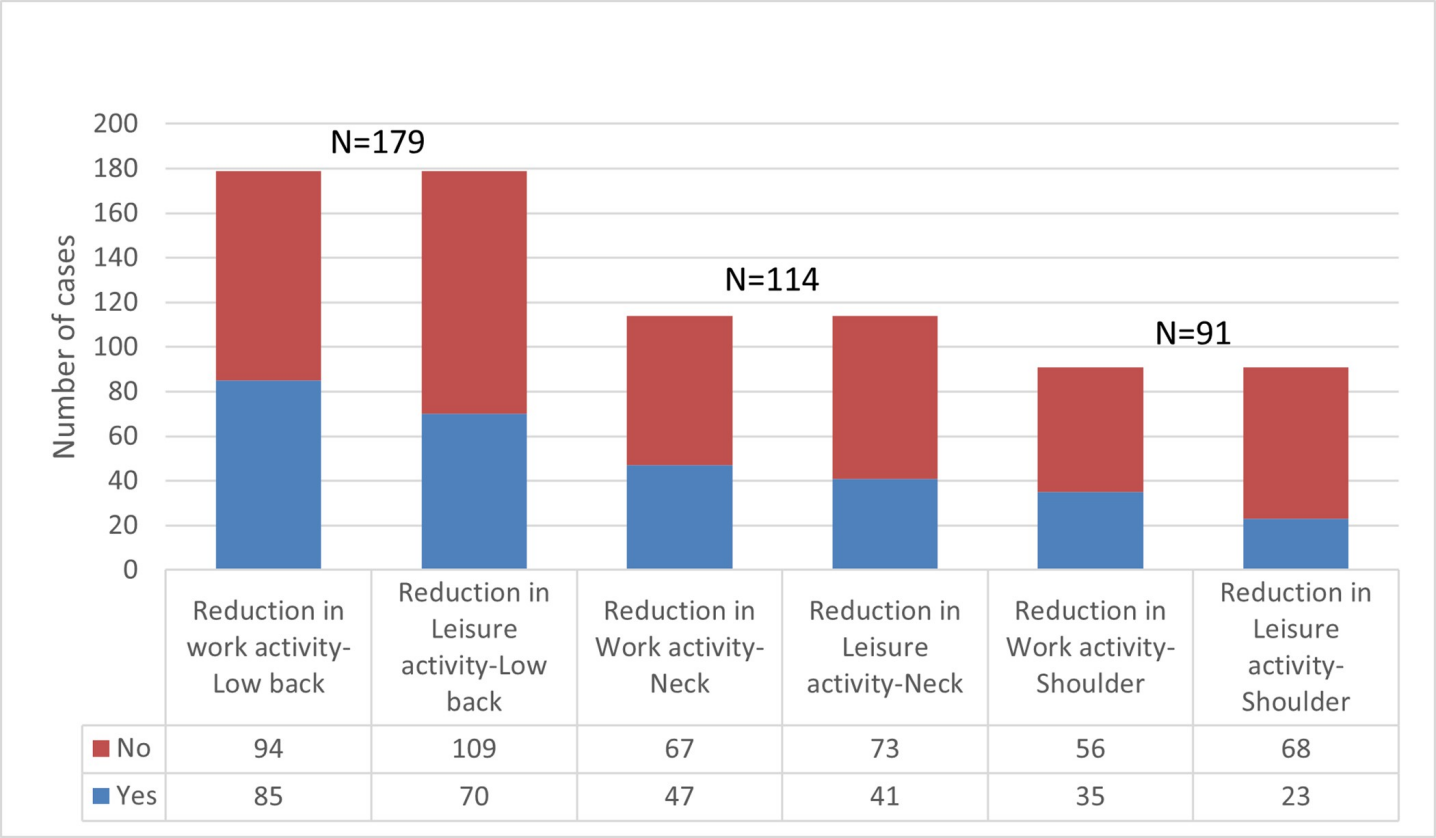
Reduction of daily activities among study participants experiencing musculoskeletal problem.

**Fig 4:**
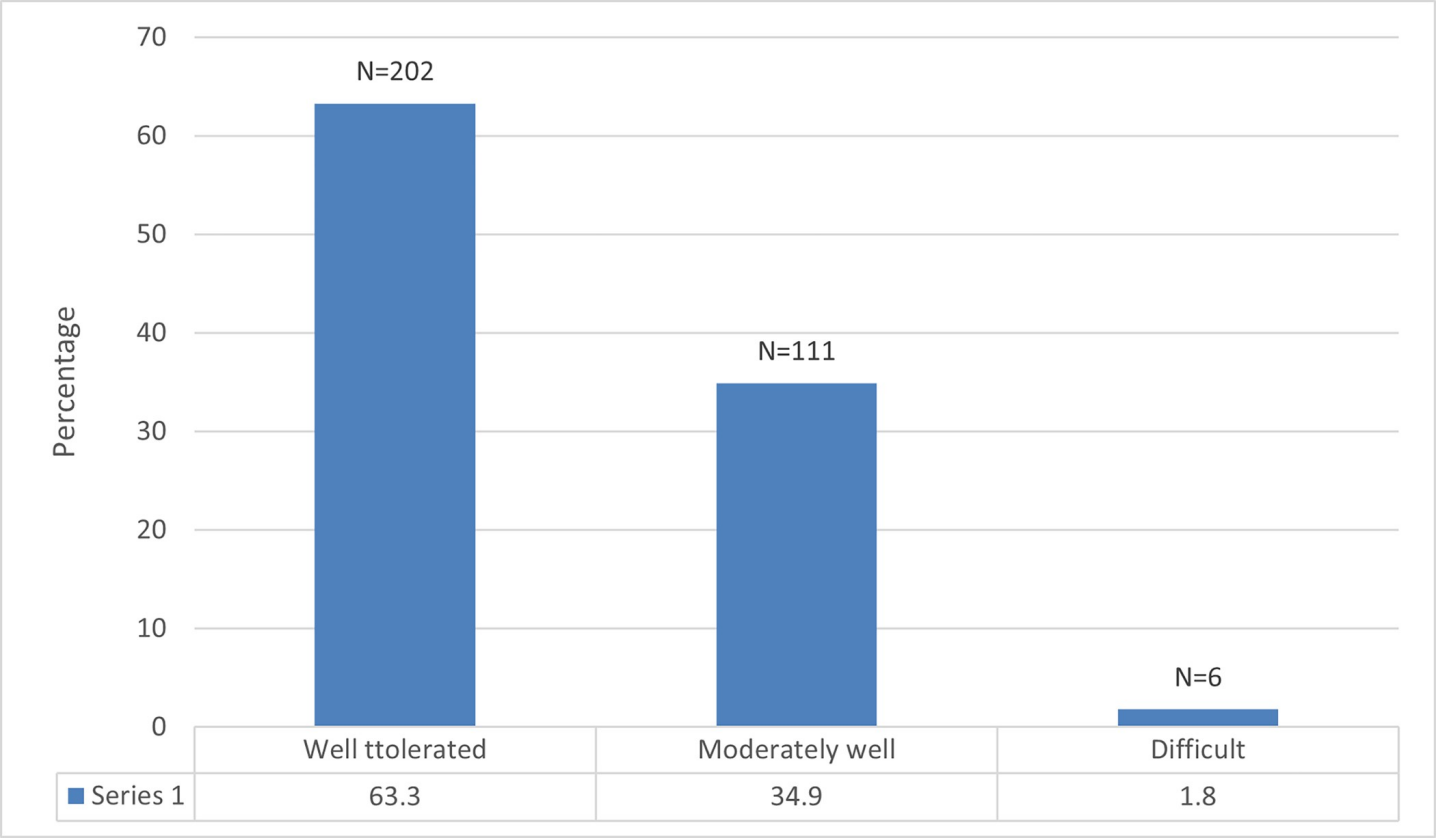
Reduction of daily activities among study participants experiencing musculoskeletal problem.

In addition, 64.8% of participants said they had more than one of these three symptoms. Fig 1 shows several sources of pain and suffering. In the previous twelve months, a high number of respondents experienced low back, neck, and shoulder pain for 1–7 days, as seen in Fig 2. As indicated in Figs 3 and 4, these issues frequently result in a reduction in both work-related and other daily activities.

### Prevalence of work-related stress

Among 319 participants, 36.7% of participants reported job-related stress. 63.3% were found to be able to efficiently handle job-related stress, 34.9% could handle it moderately, and 1.8% found it difficult to deal the stress, which must be considered.

## Discussion

Our study has observed that age, sex, use of electronics out of work, and habit of taking a break are significantly associated with computer vision syndrome. A prevalence of 89.4% (95% confidence interval [CI] (1.29, 4.89) p = 0.0070) was reported. Similarly, 80.7% of the employees had at least musculoskeletal problems, while 36.7% of the participants had work-related stress.

CVS was shown to be prevalent in 84.4% of the population, which was similar to Poudel et al. but lower than Gautam et al.(92.4%) and Shrestha et al (92.1%) [15, 21, 22]. The prevalence was found to be greater than in other Indian research [13, 14]. CVS was more prevalent in this study than in the study representing the South Asian region [18]. The prevalence of musculoskeletal symptoms in this study was found to be 80.7% which was higher than Sharma et al and Shrivastava et al. [13, 14]. Similarly, stress was present in 36.7%, which was similar to Sharma et al and Shrivastava et al. [13, 14]. The differences in prevalence are mostly attributable to differences in the work environment, workload, and population characteristics.

The existence of CVS, musculoskeletal disorders, work-related stress, and gender had no significant relationship. This finding contradicted that of P. Ranasinghe et al., who found a strong link between female gender and CVS prevalence [18]. Tired eye (63.3%), the feeling of dry eye (57.8%), headache (56.9%), and pain behind the eye (45.9%) were the main visual symptoms among the study population. Shrestha et al. also reported tired eyes (88.2%) as the most reported symptom, followed by headache (85.5%), and sore eyes (71.1%) [15]. Another study by Poudel et al. showed eyestrain (17.1%), headache (14.5%), tired eyes (14.5%), and blurred vision (8.7%) [21] as main symptoms reported. In contrast, a study by Sharma et. al. showed redness (37%), burning and/or tiredness (31.5%), headache (29.5%), pain in the eye (23.5%) as common symptoms [13]. The common symptoms among different studies were different, which may be due to the difference in population characteristics and workplace environment.

This study found that study participants using the VDT screen for more than 8 hours a day had a higher prevalence of CVS (89.4%). Sharma et al., Poudel et al, Gautam et al. also indicated that using VDT for longer hours led to a high risk for the development of CVS [13, 21, 22]. Also, not using any preventive measures like eye drops, anti-glare screens, and protective goggles were found to contribute to a higher prevalence of CVS (88.9%, 86.7%, 89.2%, respectively). Not maintaining a proper viewing distance of 40–70 cm was also found to be significantly associated with the presence of CVS; these findings were consistent with those of Poudel et al., Ranasinghe et al, Gautam et al. [18, 21, 22].

CVS symptoms can be significantly minimized by changing the work environment, including adjusting the lighting to avoid glare, maintaining a healthy distance from the computer, and taking short breaks between long breaks [23]. In our study, 15.3% study population used an anti-glare screen, and out of the 28.6%, respondents had no CVS symptoms while 84.7% didn’t use an anti-glare screen, 13.3% respondents had no CVS symptoms. In contrast to our study, a study by a Shrivastava et al. reported that out of 29% study population using an anti-glare screen, 46.6% of people didn’t have any visual problem. However, among the rest, 71%, only 27.5% were free from any visual problems. [14] According to a survey of ophthalmologists, 97.8% of doctors thought that artificial tears were the most effective treatment for CVS [24]. Similar to this study, our study reported that among 26.6% of people who used eye drops, 28.2% of those people had no symptoms of CVS. Even among eye drops, elastoviscous eye drops were shown to be more effective than regular saline solution, and herbal eye drops were found to be more successful than artificial ones in relieving symptoms [25, 26].

This improper viewing distance and spending many hours continuously in front of the VDT screen could be the reason for different musculoskeletal symptoms (80.7%). Pain in the lower back (61.5%), neck (45.2%), and shoulder (39.4%) were the most common musculoskeletal symptoms reported, which may be due to constantly sitting for a longer period. A study by Sharma et al. showed the presence of musculoskeletal symptoms in 77.5% of the study population, with pain (55%) being the most common symptoms and neck (44%) being the most affected site [13]. As concluded by Shrivastava et al., our study also showed that the frequency of having some musculoskeletal discomfort was more among the study group spending more hours per day in front of the computer (VDT screen) [14]. A study found that providing “large forearm support” combined with “ergonomic training” was an effective strategy for preventing upper body musculoskeletal diseases and reducing upper body discomfort associated with computer use [27].

Carpal tunnel syndrome is a type of nerve compression neuropathy that occurs when a nerve is compressed as it passes through the wrist [28]. In our study, 24.8% of the study population had some form of wrist pain while carpal tunnel syndrome was suspected in 11.5% of the study group. However only 5% of them tested positive for tunnels by Sharma et al. [13]. Furthermore, other research indicated that there is no significant link between computer use and carpal tunnel syndrome [28].

Similarly, work-related stress was found to be in 36.7% of the study population. Work-related stress was found to be more among employees who had started their jobs recently (0–5 years) group; (p-value<0.05) and also there was a significant relationship between work-related stress and more hours per day spent in front of the computer (VDT screen). These findings were consistent with that of Sharma et al. Males were more stressed than females, and those who had recently started the job were more stressed. The study population had minimal to mild depression, according to Zung’s self-rating depression scale and Hamilton’s depression rating scale (8% and 6%, respectively) [13].

### Strengths and limitations

The main strength of this study is that this is the first study to analyze the visual problems, musculoskeletal, and stress related problems among visual display terminal users in Nepal through validated questionnaire. This provides the reliability to our study. Our research is significant because, in this age of technology and digitalization, we often forget that our bodies require proper posture and timing when using computers and the internet. As a result, it will raise community awareness and assist organizations in designing appropriate work ergonomics. Improved internet and computer education and training has a big impact on computer users’ posture care. Replacing a sedentary lifestyle with activities that include good physical activity and a healthy diet can help to alleviate these issues.

The study due to the fact that this was a cross-sectional study with a self-administered questionnaire, social desirability and recollection bias could have been introduced. Individuals who are extremely ill and unable to work may be excluded from the data collection process. Except for hazy vision, the majority of eye problems were self-reported. Medical examinations will be used by future researchers to address eye issues. The sample size of the study was small and hence the result of this study may not represent work related musculoskeletal disorders of all IT workers in Nepal. Another limitation was that the questions were based on respondents’ memories of their experiences over the past months, which could lead to memory bias and overstatements.

## Conclusion

CVS and musculoskeletal difficulties were found in a substantial proportion of VDT screen users in this cross-sectional investigation. There was no correlation between gender and visual complaints, musculoskeletal problems, or stress. CVS was found to be substantially related to not employing preventive measures, working longer hours, and having an incorrect viewing distance. Workplace stress was shown to be higher among those who had recently started their jobs. Working for fewer hours at a time, taking breaks, and maintaining proper viewing distance would help to reduce the musculoskeletal symptoms and visual problems. Proper distribution of workload, supportive colleagues, and supportive feedback would reduce work-related stress. A similar study, on a larger scale, would help to find out the actual situation in the nation and will help in making institutional plans and policies to prevent it. The findings of this study will help ophthalmic experts, health care providers, and other training institutions plan, design, and revise training curricula to improve employees’ knowledge and awareness of CVS.

## Supporting information

S1 File Questionnaire used for the survey. (DOCX)
