# Supplementary material for: Computer vision syndrome, musculoskeletal, and stress-related problems among visual display terminal users in Nepal
Source: PLoS One. 2022 Jul 19;17(7):e0268356. doi: 10.1371/journal.pone.0268356 (PMC9295968; doi:10.1371/journal.pone.0268356)
Supplement: S1 File — (DOCX) [file pone.0268356.s001.docx]

Supplemental information 1: questionnaire used for the survey

**Pro forma sheet**

Name:-

Age:-

Gender:-

How much year and month have you been doing your present work?

On average, how much hour do you work per day?

Height:-

Are you Left handed

Right handed

Use of anti-glare screen:-

Use of artificial tear drops:-

Visual Acuity:- Right eye:- Left eye:-

Schirmer test:- Right eye:- Left eye:-

a) VISUAL SYMPTOMS:- Tribhuvan University, Institute of Medicine, Visual Problems Among Video Display Terminal (VDT) Users in Nepal. Research Questionnaire

Scale:-

None Very mild Mild Moderate intense very intense

0 1 2 3 4 5

| Symptoms | None | Very mild | Mild | Moderate | Intense | Very intense |
| --- | --- | --- | --- | --- | --- | --- |
| Watery eye |  |  |  |  |  |  |
| Dry eye |  |  |  |  |  |  |
| Pain behind eye |  |  |  |  |  |  |
| Itchy eye |  |  |  |  |  |  |
| Tierd eye |  |  |  |  |  |  |
| Eye redness |  |  |  |  |  |  |
| Blurred vision |  |  |  |  |  |  |
| Burning eye |  |  |  |  |  |  |
| Head ache |  |  |  |  |  |  |

b) Nordic Questionnaire:-

| Trouble with locomotive organs | | |
| --- | --- | --- |
| Have you at any time during the last 12 months had trouble (ache, pain, discomfort) in | To be answered only by those who have had trouble | |
|  | Have you at any time during the last 12 months been prevented from doing your normal work because of trouble? | Have you had trouble at any time during the last 7 days? |
| Neck  No  Yes | No Yes | No Yes |
| Shoulder  No Yes, in the right shoulder  Yes, in the left shoulder  Yes, in the both shoulder | No Yes | No Yes |
| Elbows  No Yes, in the right elbow  Yes, in the left elbow  Yes, in the both elbow | No Yes | No Yes |
| Wrists/ Hands  No Yes, in the right wrist/hand  Yes, in the left wrist/hand  Yes, in the both wrist/hand | No Yes | No Yes |
| Upper Back  No  Yes | No Yes | No Yes |
| Lower Back  No  Yes | No Yes | No Yes |
| One or Both hips/thigh  No  Yes | No Yes | No Yes |
| One or Both knee  No  Yes | No Yes | No Yes |
| One of Both ankles/feet  No  Yes | No Yes | No Yes |

LOW BACK

1. Have you ever had low back trouble (ache, pain or discomfort)?

No  Yes

If you answered **NO** in question No. 1, do not answer question 2-8

1. Have you ever been hospitalized because of low back trouble?

No  Yes

1. Have you ever had to change jobs or duties because of low back trouble?

No  Yes

1. What is the total length of time that you have had low back trouble during the last 12 months?

0 days

1-7 days

8-30 days

More than 30 days but not every day

Every day

If you answered **0 days** to question 4, do not answer questions 5-8

1. Has low back trouble caused you to reduce your aciviy during the last 12 months?
2. Work activity?  No  Yes
3. Leisure activity?  No  Yes
4. What is the total length of time that low back trouble has prevented you from doing your normal work during last 12 months?

0 days

1-7 days

8-30 days

More than 30 days

1. Have you been seen by a doctor, physiotherapist because of low back trouble during last 12 months?

No  Yes

1. Have you had low back trouble at any time during the last 7 days?

No  Yes

NECK

1. Have you ever had neck trouble (ache, pain or discomfort)?

No  Yes

If you answered **NO** in question No. 1, do not answer question 2-8

1. Have you ever hurt your neck in an accident?

No  Yes

1. Have you ever had to change jobs or duties because of neck trouble?

No  Yes

1. What is the total length of time that you have had neck trouble during the last 12 months?

0 days

1-7 days

8-30 days

More than 30 days but not every day

Every day

If you answered **0 days** to question 4, do not answer questions 5-8

1. Has neck trouble caused you to reduce your aciviy during the last 12 months?
2. Work activity?  No  Yes
3. Leisure activity?  No  Yes
4. What is the total length of time that neck trouble has prevented you from doing your normal work during last 12 months?

0 days

1-7 days

8-30 days

More than 30 days

1. Have you been seen by a doctor, physiotherapist because of low back trouble during last 12 months?

No  Yes

1. Have you had low back trouble at any time during the last 7 days?

No  Yes

SHOULDER

1. Have you ever had shoulder trouble (ache, pain or discomfort)?

No  Yes

If you answered **NO** in question No. 1, do not answer question 2-8

1. Have you ever hurt your shoulder in an accident?

No  Yes

1. Have you ever had to change jobs or duties because of shoulder trouble?

No  Yes

1. Have you had shoulder trouble during the last 12 months?

No  Yes, in my right shoulder

Yes, in my left shoulder

Yes, in my both shoulder

If you answered **NO** to question 4, do not answer questions 5-9.

1. What is the total length of time that you have had shoulder trouble during the last 12 months?

0 days

1-7 days

8-30 days

More than 30 days but not every day

Every day

1. Has shoulder trouble caused you to reduce your aciviy during the last 12 months?
2. Work activity?  No  Yes
3. Leisure activity?  No  Yes
4. What is the total length of time that shoulder trouble has prevented you from doing your normal work during last 12 months?

0 days

1-7 days

8-30 days

More than 30 days

1. Have you been seen by a doctor, physiotherapist because of low back trouble during last 12 months?

No  Yes

1. Have you had shoulder trouble at any time during the last 7 days?

No  Yes

c) Work related stress

**1.** I am clear what is expected of me at work

Never(1) seldom(2) sometimes(3) often (4) always(5)

**2.** I can decide when to take a break

Never(1) seldom(2) sometimes(3) often (4) always(5)

**3.** Different groups at work demand things from me that are hard to combine

Never(1) seldom(2) sometimes(3) often (4) always(5)

**4.** I know how to go about getting my job done

Never(1) seldom(2) sometimes(3) often (4) always(5)

**5.** I am subject to personal harassment in the form of unkind words or behaviour

Never(5) seldom(4) sometimes(3) often (2) always(1)

**6.** I have unachievable deadlines

Never(5) seldom(4) sometimes(3) often (2) always(1)

**7.** If work gets difficult, my colleagues will help me

Never(1) seldom(2) sometimes(3) often (4) always(5)

**8.** I am given supportive feedback on the work I do

Never(1) seldom(2) sometimes(3) often (4) always(5)

**9.** I have to work very intensively

Never(5) seldom(4) sometimes(3) often (2) always(1)

**10.** I have a say in my own work speed

Never(1) seldom(2) sometimes(3) often (4) always(5)

**11.** I am clear what my duties and responsibilities are

Never(1) seldom(2) sometimes(3) often (4) always(5)

**12.** I have to neglect some tasks because I have too much to do

Never(5) seldom(4) sometimes(3) often (2) always(1)

**13.** I am clear about the goals and objectives for my department

Never(1) seldom(2) sometimes(3) often (4) always(5)

**14.** There is friction or anger between colleagues

Never(5) seldom(4) sometimes(3) often (2) always(1)

**15.** I have a choice in deciding how I do my work

Never(1) seldom(2) sometimes(3) often (4) always(5)

**16.** I am unable to take sufficient breaks

Never(5) seldom(4) sometimes(3) often (2) always(1)

**17.** I understand how my work fits into the overall aim of the organisation

Never(1) seldom(2) sometimes(3) often (4) always(5)

**18.** I am pressured to work long hours

Never(5) seldom(4) sometimes(3) often (2) always(1)

**19.** I have a choice in deciding what I do at work

Never(1) seldom(2) sometimes(3) often (4) always(5)

**20.** I have to work very fast

Never(5) seldom(4) sometimes(3) often (2) always(1)

**21.** I am subject to bullying at work

Never(5) seldom(4) sometimes(3) often (2) always(1)

**22.** I am aware of others being subject to bullying at work

Never(5) seldom(4) sometimes(3) often (2) always(1)

**23.** If I were aware of bullying I would feel able to challenge it

Never(1) seldom(2) sometimes(3) often (4) always(5)

**24.** If I reported bullying, I would be confident that it would be stopped

Never(1) seldom(2) sometimes(3) often (4) always(5)

**25.** I have unrealistic time pressures

Never(5) seldom(4) sometimes(3) often (2) always(1)

**26.** I can rely on my line manager to help me out with a work problem

Never(1) seldom(2) sometimes(3) often (4) always(5)

**27.** I get help and support I need from colleagues

Strongly disagree(1) disagree(2) Neutral(3) Agree(4) strongly agree(5)

**28.** I have some say over the way I work

Strongly disagree(1) disagree(2) Neutral(3) Agree(4) strongly agree(5)

**29.** I have sufficient opportunities to question managers about change at work

Strongly disagree(1) disagree(2) Neutral(3) Agree(4) strongly agree(5)

**30.** I receive the respect at work I deserve from my colleagues

Strongly disagree(1) disagree(2) Neutral(3) Agree(4) strongly agree(5)

**31.** Staff are always consulted about change at work

Strongly disagree(1) disagree(2) Neutral(3) Agree(4) strongly agree(5)

**32.** I can talk to my line manager about something that has upset or annoyed me about work

Strongly disagree(1) disagree(2) Neutral(3) Agree(4) strongly agree(5)

**33.** My working time can be flexible

Strongly disagree(1) disagree(2) Neutral(3) Agree(4) strongly agree(5)

**34.** My working location can be flexible (subject to business constraints)

Strongly disagree(1) disagree(2) Neutral(3) Agree(4) strongly agree(5)

**35.** My colleagues are willing to listen to my work-related problems

Strongly disagree(1) disagree(2) Neutral(3) Agree(4) strongly agree(5)

**36.** When changes are made at work, I am clear how they will work out in practice

Strongly disagree(1) disagree(2) Neutral(3) Agree(4) strongly agree(5)

**37.** I am supported through emotionally demanding work

Strongly disagree(1) disagree(2) Neutral(3) Agree(4) strongly agree(5)

**38.** Relationships at work are strained

Strongly disagree(5) disagree(4) Neutral(3) Agree(2) strongly agree(1)

**39.** My line manager encourages me at work

Strongly disagree(1) disagree(2) Neutral(3) Agree(4) strongly agree(5)
